# Impacts of Menstruation, Community Type, and an Oral Yeast Probiotic on the Vaginal Microbiome

**DOI:** 10.1128/msphere.00239-22

**Published:** 2022-09-14

**Authors:** E. Oerlemans, S. Ahannach, S. Wittouck, E. Dehay, I. De Boeck, N. Ballet, B. Rodriguez, I. Tuyaerts, S. Lebeer

**Affiliations:** a University of Antwerp, Department of Bioscience Engineering, Antwerp, Belgium; b Gnosis by Lesaffre, Lesaffre Group, Marcq-en-Baroeul, France; c Lesaffre International, Lesaffre Group, Marcq-en-Baroeul, France; University of California, Davis

**Keywords:** *Saccharomyces cerevisiae*, intervention study, probiotics, vaginal microbiota, yeasts

## Abstract

A healthy state of the vaginal microbiome can prevent vaginal disease and promote successful fertilization and healthy pregnancies. Little is known about the stability of the vaginal microbiome and the influence of factors such as diet and probiotics. While less explored, yeast probiotics have an interesting potential because of their immunomodulatory and pathogen inhibition capacities. In this study, we investigated the impact of the oral yeast probiotic Saccharomyces cerevisiae CNCM I-3856 on the vaginal microbiomes of 52 healthy women using 16S and internal transcribed spacer (ITS) amplicon sequencing and quantitative PCR (qPCR). The vaginal fungal loads remained low, even after oral yeast supplementation, complicating the analysis of the vaginal mycobiome. Lactobacillus crispatus and Lactobacillus iners were the most dominant species in our study population and were found to codominate in 23% of the baseline samples. *Bifidobacterium*, Streptococcus, and *Prevotella* were also frequently found. The microbiome profiles were dynamic: 69% of women showed a shift in the dominant community members at least once during the 42-day sampling period. In addition, lower *Lactobacillus* abundances were observed at the time points after menstruation. Higher relative abundances of *Lactobacillus* with more L. iners*-*dominated samples and a trend toward lower relative abundances of *Prevotella* were observed in the probiotic group, but analyses of the effects of the yeast probiotic were complicated by differences already present at the onset of the study. Thus, our findings especially highlighted that the impact of menstruation and the stratification of women based on the dominant vaginal taxa before randomization and inclusion is important for future research: while the impact of the yeast probiotic on vaginal microbiome in healthy women was limited.

**IMPORTANCE** How to define and promote a healthy state of the vaginal microbiome is not well understood. Knowledge of which underlying factors shape the microbial community composition of the vagina and how to modulate them will contribute to vaginal disease prevention and improve fertility. Here, we found that taking the menstrual cycle into account when designing a microbiome study is highly recommended: menstruation also showed to be poses an interesting time point for intervention because of the drop in the abundance of L. crispatus. Furthermore, the early stratification of groups (e.g., placebo versus treatment) according to the dominant taxa can be of high added value since menstruation impacts vaginal taxa differently, i.e., *L. iners* remains stable, in contrast to *L. crispatus*.

## INTRODUCTION

For women, the vaginal microbiome is undoubtedly one of the most important microbial communities in their bodies, impacting their general health and well-being and those of their children and partners (1–3). In healthy women, the microbiome is generally dominated by one of four *Lactobacillus* species, L. crispatus, L. iners, L. gasseri, or L. jensenii (4–7), all of which have been linked to vaginal health to various degrees (2). Additionally, recent insights highlight modules of bacteria cooccurring in the vaginal microbiome (6) and indicate the beneficial activity of multimicrobial consortia (8). Besides *Lactobacillus-*dominated profiles, other typical vaginal microbiome profiles have also been described, such as profiles with a high bacterial richness and a high relative abundance of anaerobes, including *Gardnerella*, *Fannyhessea* (recent reclassification of *Atopobium* [9]), *Dialister*, and *Prevotella* (7). Vaginal microbiomes rich in Streptococcus, Staphylococcus, *Bifidobacterium*, *Prevotella*, *Enterococcus*, Escherichia/*Shigella*, Haemophilus, or Campylobacter have also been observed (10–14).

The influence of these bacterial taxa, which include potential pathobionts, on vaginal health is not well understood, but associations between vaginal health or, conversely, vaginal conditions and specific microbiome profiles have been suggested (11, 15–17). Evidence on the mechanisms underlying how beneficial microbes such as lactobacilli promote health and what environmental and host factors drive the vaginal microbiome toward a certain community composition is lacking. Dietary patterns, the natural hormonal cycle, contraceptives, probiotics, and vitamin supplements have already been reported to have some impact (18) and are also being studied in our citizen-science project Isala (https://isala.be/en).

To gain more mechanistic insights, microbiome modulation studies, such as through the application of (potential) probiotics, are a valuable tool. Studies using probiotics for vaginal conditions and microbiome modulation have been performed with variable success (19–24). However, the successful application of probiotics depends on many variables, including the chosen strain, administration route, and timing of supplementation. Many probiotics belong to the *Lactobacillaceae* family, and only a few vaginally isolated probiotic strains are used. Yeast probiotics are even less explored in applications targeting the vaginal microbiome (25) because the abundance and prevalence of yeasts under healthy conditions are much lower than those of lactobacilli (26). Nonetheless, yeasts such as Saccharomyces cerevisiae have strong immunological and microbiological potential, especially against infections with pathogenic strains from the closely related genus *Candida*, which occur commonly (25, 27–30). Yeast probiotics are also currently being explored for other vaginal conditions such as bacterial vaginosis, with a role for *Saccharomyce*s spp. in inhibiting Gardnerella vaginalis (31). Yeast probiotics have the advantage of having a long history of use in food and nutritional supplements, and they can be applied in a safe and cost-effective way (25). Furthermore, yeasts avoid the risk of the transfer of antibiotic resistance genes and can be administered with antibiotic treatments, which is highly relevant considering the current treatment of bacterial vaginal infections (25, 31).

The administration route is a key factor influencing the efficacy of a probiotic. Vaginal application has the main advantage of delivering the microorganisms directly, in high concentrations, to the location where their activity is necessary. Nonetheless, oral application is accepted by more women, improving patient compliance. Oral application might especially be favored over topical application during menstruation. Furthermore, gut-to-vaginal transmission appears possible, as a direct connection exists between food, the gut, and the vagina, with the fecal microbiota acting as a potential source of the vaginal microbiota (32, 33). Additionally, even if no transmission occurs, possible immune modulation by probiotics in the gut may also have more distant effects (34). The gut is a key environment for interactions between the microbiota and the immune system, e.g., influencing T cell populations such as regulatory T cells, and is an important point of entry for microbial metabolites such as short-chain fatty acids to reach the bloodstream, also allowing distant systemic effects across the whole body. In this way, microbes in the gut can also improve (or exacerbate) symptoms of non-gut-related conditions, as previously reviewed (35).

In this study, one of the best-characterized yeast probiotics, Saccharomyces cerevisiae CNCM I-3856 (36), which has been shown to be effective against *Candida* and bacterial vaginosis *in vitro* and in animal models (25), was investigated. We evaluated whether oral supplementation was able to impact the bacterial and fungal vaginal microbiomes during and after a 4-week intervention period and throughout specific time points in the menstrual cycle.

## RESULTS

### Prevalence and abundance of commensal yeasts versus lactobacilli.

**FIG 1:**
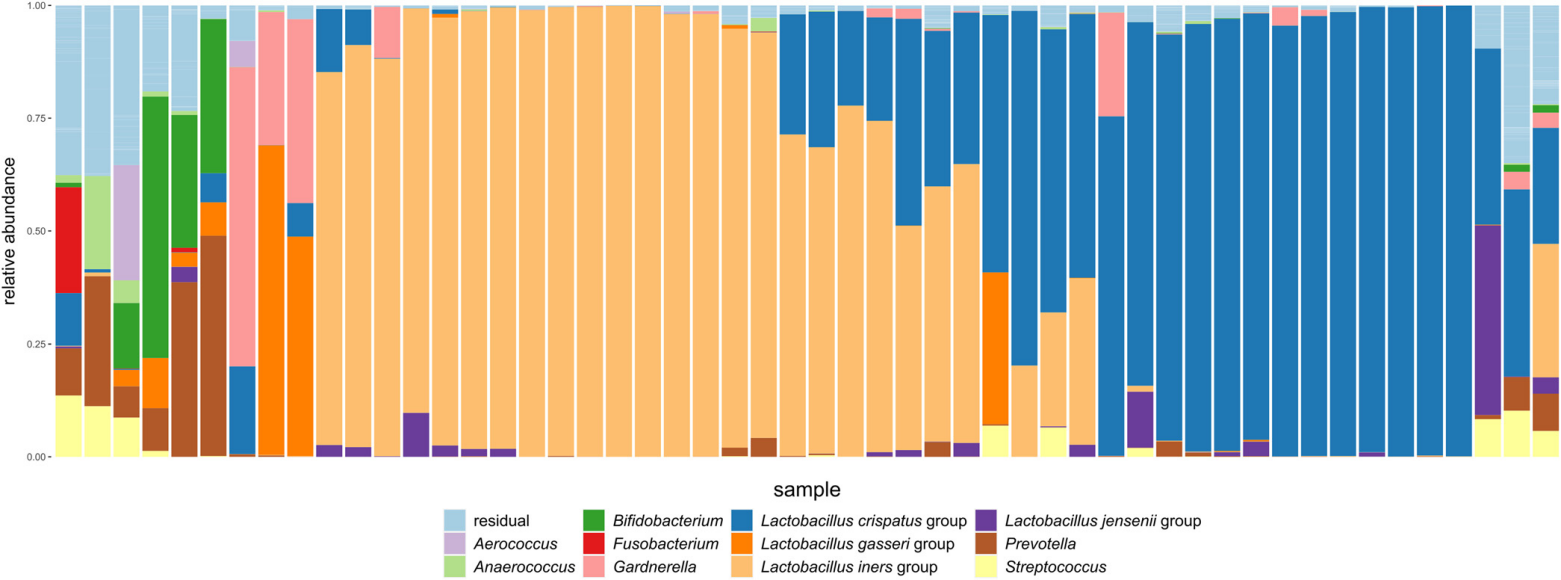
Taxonomic classification of bacterial communities of baseline samples at visit 1. The relative abundances of the top 11 most abundant taxa at the genus level, or the subgenus level for the *Lactobacillus* genus, are indicated. All other taxa are grouped as “residual.” Different samples are presented along the *x* axis, with each stacked bar representing the baseline sample from one woman. Samples are ordered according to similarity by minimizing the Bray-Curtis distance between neighboring samples.

Fifty-two healthy women were included in the study (see Materials and Methods). To have an idea of the composition and relative importance of endogenous yeasts and bacteria in the vaginal microbial communities of these women, the samples were investigated through 16S rRNA V4 sequencing (for bacteria), internal transcribed spacer 2 (ITS2) sequencing (for yeast), and quantitative PCR (qPCR) (for more semiquantitative measurements). We first evaluated the microbial communities of the samples at visit 1, which were taken prior to the administration of the probiotic or placebo, to obtain the vaginal community compositions at baseline without the influence of the intervention. qPCR analyses confirmed relatively high concentrations of lactobacilli, with an average estimated concentration of 7.5 × 10^9^ CFU/mL (median of 6.8 × 10^9^ CFU/mL; range, 8.78 × 10^7^ CFU/mL to 4.06 × 10^10^ CFU/mL). Our 16S data showed that these lactobacilli constituted almost the entire community, with *Lactobacillus* taxa (according to the new taxonomic classification [37]) amounting to an average relative abundance of 81.8% (median of 97.4%), as indicated in Fig. 1. At a lower level of diversity, more precisely, at the amplicon sequence variant (ASV) level, we found several ASVs belonging to the *Lactobacillus* genus, with 6 *Lactobacillus* ASVs being ranked among the 11 most abundant ASVs over all genera. These ASVs were annotated as belonging to the Lactobacillus crispatus group (3 ASVs [*Lactobacillus* ASVs 2, 4, and 6]), the Lactobacillus iners group (*Lactobacillus* ASV 1), the Lactobacillus gasseri group (*Lactobacillus* ASV 3), and the Lactobacillus jensenii group (*Lactobacillus* ASV 5) (Fig. 1). Among the samples from the first visit, most were dominated by the L. crispatus group (≥40% relative abundance in baseline samples from 18/52 women), the *L. iners* group (≥40% relative abundance in baseline samples from 24/52 women), or both groups (baseline samples from 12 women with abundances of each taxon of ≥10% and their combined relative abundance of ≥50%, 3 with higher abundances of the L. crispatus group and 9 with higher abundances of the *L. iners* group), as shown in Fig. 1. Two baseline samples were dominated by L. gasseri group ASVs (≥40% relative abundance), while baseline samples from two other women also contained this taxon at high relative abundances (>10%). L. jensenii ASVs were dominant in one baseline sample (>40% relative abundance) and exceeded 10% relative abundance in one other baseline sample. In the baseline samples from 7 women, *Lactobacillus* relative abundances remained below 40%. *Gardnerella* was dominant (≥40%) in two baseline samples and highly abundant in three others (>10% relative abundance). Other abundant taxa were *Bifidobacterium* (*≥*40% in 1 sample and ≥10% in 4 baseline samples), *Prevotella* (>40% in 1 and ≥10% in 4 baseline samples), and Streptococcus (≥10% in 3 baseline samples). Of note, a typical highly diverse bacterial vaginosis-like profile dominated by the genera *Gardnerella*, *Prevotella*, and *Atopobium*, among others, was not detected in our study cohort of healthy women. Attempts to amplify the ITS2 region, often used to study the fungal community in a fashion similar to that for 16S rRNA gene sequencing for bacterial communities, such as in our previous work (24), yielded largely unsuccessful PCR results, with only a few samples (baseline visit, 0/52; entire study, 6/301) indicating some amplification (PCR product yields higher than twice the highest concentration of the negative control). Dedicated yeast culture was also performed, which confirmed the low endogenous levels of fungi and yeast, with only 1 sample out of 52 showing a positive culture result for *Candida* spp., by using a chromogenic medium (not further identified to the species level).

### Fluctuations of the vaginal microbiome throughout the menstrual cycle.

**FIG 2:**
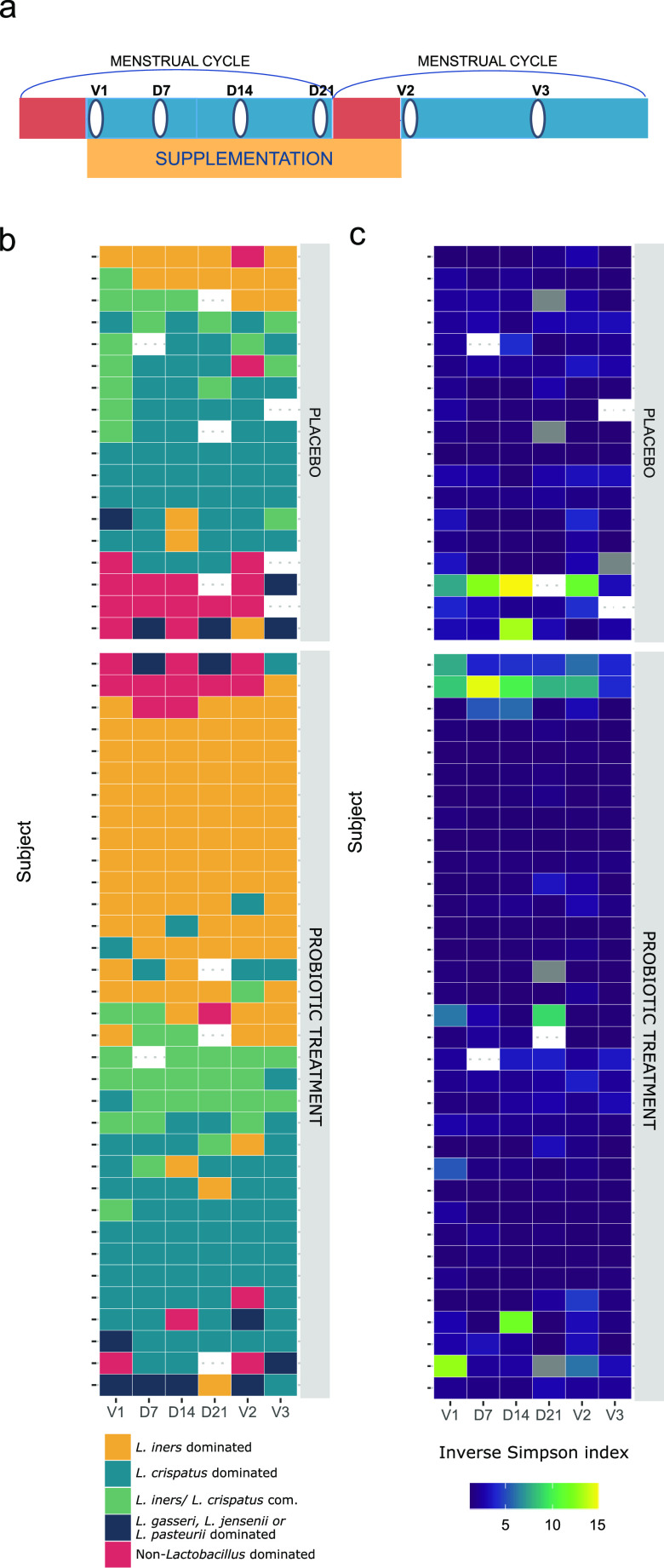
Analysis of the dominant taxa (or pairs of taxa) and alpha diversity of samples by participant and study group. (a) Schematic showing a simplified timeline of the study. Red bars indicate menstruation, the yellow bar indicates supplementation with the study medication/placebo, and white ovals indicate a sampling time point. (b) The dominant taxa (or pairs of taxa) of each sample ordered by study time point (*x* axis) and participant (*y* axis). Colors indicate to which type a certain sample belonged. (c) Alpha diversity of each sample ordered by study time point (*x* axis) and participant (*y* axis), based on the inverse Simpson metric. com., combined.

Without prior knowledge of their vaginal microbiomes, 52 women had been randomly allocated to receive a daily supplement that was either an oral placebo (*n* = 18) or the oral probiotic Saccharomyces cerevisiae CNCM I-3856 (*n* = 34) (at a daily dose of 500 mg [*n* = 17] or 1,000 mg [*n* = 17]) (36). For the current analyses, treatment groups were combined as the dose received did not seem to impact gut-vaginal translocation (36). Vaginal samples were collected as described in Materials and Methods: three swabs were collected during a visit to the study center (time points V1, V2, and V3), and three swabs were collected by self-sampling (time points D7, D14, and D21). Supplementation with the probiotic or placebo started immediately after menstruation and continued over a 4-week period (Fig. 2a; see also Fig. S1 in the supplemental material). Since the intervention started just after menstruation and only women with a menstrual cycle length of 28 (±2) days were included, samples collected at the same time point from different women corresponded to similar phases of the menstrual cycle (Fig. 2a; Fig. S1). Because menstruation was postulated to have a strong impact on the vaginal community (38), regardless of oral yeast supplementation in these healthy women, we first substantiated the effect of menstruation, without discriminating between the placebo and probiotic groups.

10.1128/msphere.00239-22.1 FIG S1 Detailed schedule of the study design as reported previously by A. Decherf, E. Dehay, M. Boyer, M. Clément-Ziza, et al. (Nutrients 12:2211, 2020, https://doi.org/10.3390/nu12082211). Download FIG S1, TIF file, 1.7 MB. Copyright © 2022 Oerlemans et al. 2022 Oerlemans et al. https://creativecommons.org/licenses/by/4.0/ This content is distributed under the terms of the Creative Commons Attribution 4.0 International license.

To investigate the stability of the vaginal microbiome, we grouped samples according to the most dominant taxa (Fig. 2b; see also Materials and Methods), more precisely, (i) L. crispatus group-dominated samples; (ii) *L. iners* group-dominated samples; (iii) samples with L. crispatus and *L. iners* codominating (each at >10% and together at >50% relative abundances); (iv) L. gasseri group-, L. jensenii group-, or L. pasteurii group-dominated samples; and (v) non*-Lactobacillus*-dominated samples. L. gasseri, L. jensenii, and *L. pasteurii* group-dominated samples (10, 2, and 2 samples, respectively) were grouped to limit the number of groups. As expected, we observed the highest alpha diversity (mean inverse Simpson index, 6.99) for the non-*Lactobacillus*-dominated profiles (Fig. 2c). We found that the vaginal samples taken at different time points across two menstrual cycles had the same dominant taxon (or taxon pair) in 16 of 52 women (31%). In the other 36 women (69%), one or more samples showed another dominant taxon compared to the majority of their samples. More precisely, 33%, 21%, and 15% of the women showed 1, 2, or ≥3 shifts in their dominant community member (Fig. 2b). It is important to note that shifts from the codominating L. crispatus group-*L. iners* group to either the L. crispatus group or the *L. iners* group (or vice versa) were the most common in our study set. Shifts occurred at any time point, but V1 and V2 (both time points after menstruation) seemed to be more likely to diverge from the other samples.

**FIG 3:**
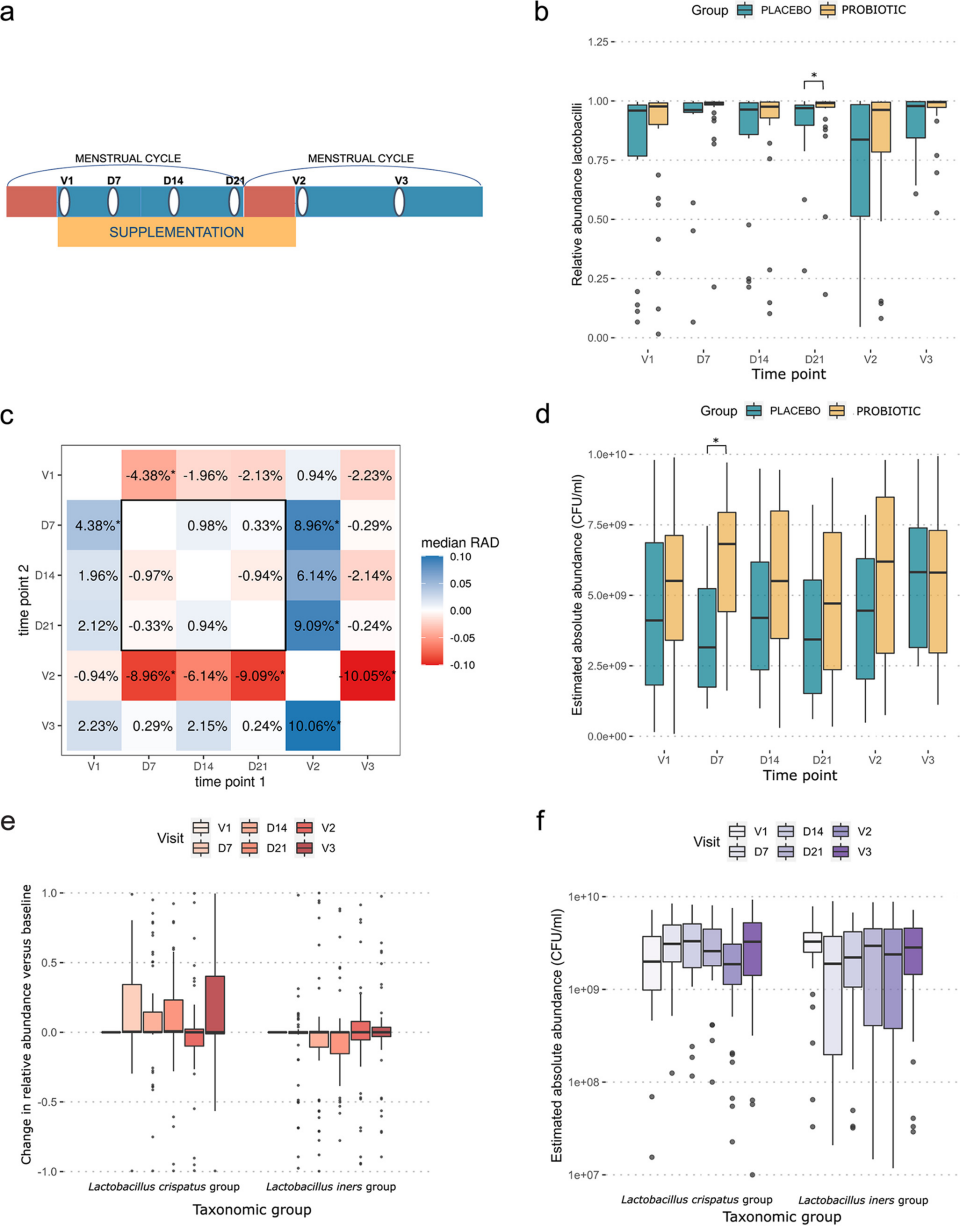
Abundances of lactobacilli over the course of the study. (a) Schematic showing a simplified timeline of the study. Red bars indicate menstruation, the yellow bar indicates supplementation with the study medication/placebo, and white ovals indicate a sampling time point. (b) Box plot showing tje relative abundances of the *Lactobacillus* genus for the placebo group (blue) (left) and the two probiotic groups combined (treatment) (yellow) (right). (c) The relative abundances of lactobacilli were compared for all combinations of time points. Each cell compares two time points and contains the median relative abundance difference (RAD) between the time points. A positive value indicates that the participants had higher *Lactobacillus* abundances at time point 2 (*y* axis) than at time point 1 (*x* axis). (d) Box plot of estimated absolute abundances of the *Lactobacillus* genus for the placebo group (blue) (left) and the two probiotic groups combined (treatment) (yellow) (right). (e) Box plot showing changes in the relative abundances of L. crispatus group ASVs (left) and *L. iners* group ASVs (right), combined for the placebo and probiotic groups. This was calculated by subtracting the relative abundance of the specific taxon in the V1 sample from the same participant from the relative abundance of the sample. (f) Box plot indicating the estimated absolute abundances of L. crispatus (left) and *L. iners* (right) in the samples. The red (e) and purple (f) shades indicate the time points at which the samples were collected. *P < 0.05.

As the *Lactobacillus* genus plays a principal role in the vaginal microbial community, we subsequently evaluated the relative and absolute abundances of lactobacilli throughout the study period (Fig. 3). We observed that the samples collected at V1 (baseline) and especially V2 (after 4 weeks of supplementation), both just after menstruation (Fig. 3a), showed lower relative abundances of lactobacilli than samples collected at the other time points (D7, D14, D21, and V3) (Fig. 3b). For V1 (baseline), this difference was small (1.96% to 4.38% median reduction in lactobacilli compared to the other time points, not taking V2 into account) (Fig. 3c) and was significant only for the comparison with time point D7 (*P* < 0.05). For visit 2 (after 4 weeks of supplementation), the difference was larger (6.76% to 10.05% reduction in lactobacilli compared to the other time points, not taking V1 into account) as well as statistically significant for three out of four time points (*P* < 0.05), with time point D14 being the exception. A similar trend (low relative abundances at V1 and V2 and higher relative abundances at the other time points) was observed for the cumulative relative abundance of L. crispatus group ASVs (average relative abundances of 35.3% for V1 and V2 and 46.5% for the other time points) but not for the *L. iners* group (Fig. 3e) (average relative abundances of 38.9% for V1 and V2 and 36.8% for the other time points). When using primers specific for L. crispatus, the absolute concentrations estimated by qPCR were in line with the fluctuations in the relative abundances (Fig. 3f). Overall, the absolute qPCR concentrations with primers specific for *L. iners* also confirmed the 16S amplicon data showing that the abundance of this taxon remained relatively stable (Fig. 3f).

### Differences in bacterial communities between the probiotic and placebo groups.

**FIG 4:**
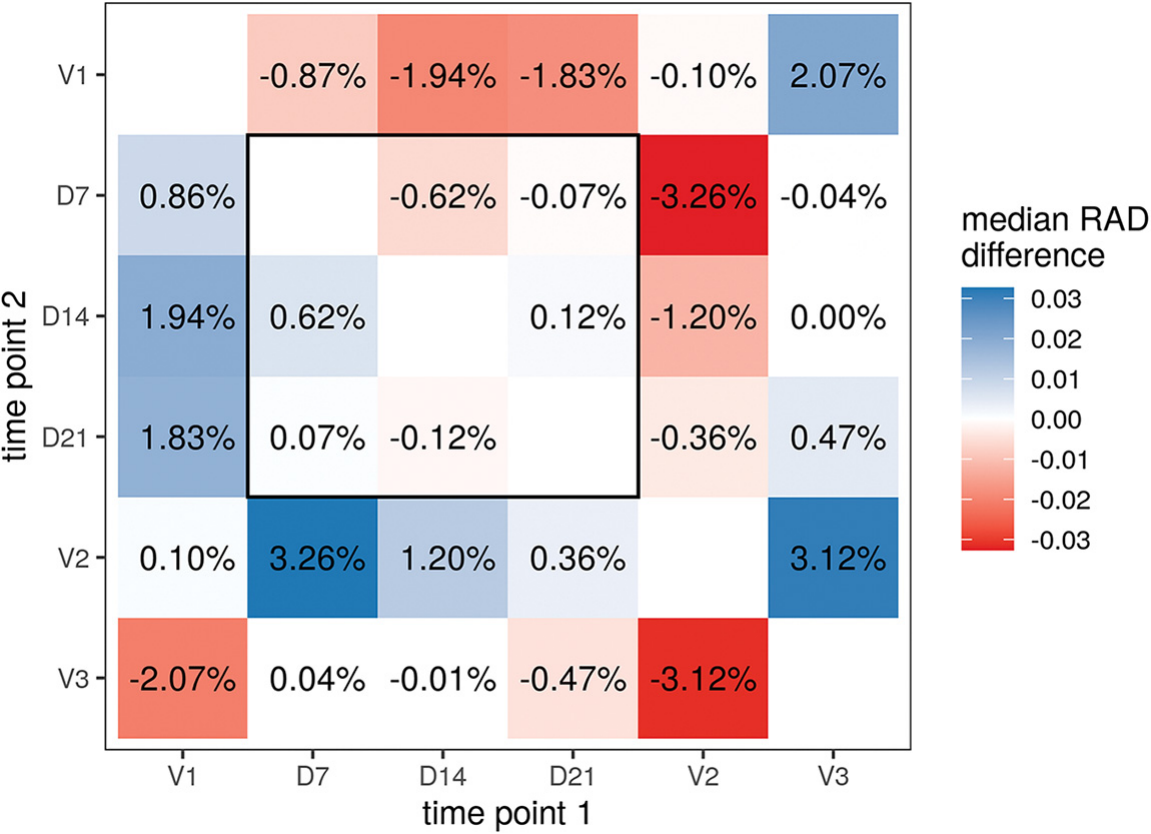
Impact of probiotic supplementation on the evolution of the abundances of lactobacilli throughout the study. Shown are differential RAD values of *Lactobacillus* between treatments. Each cell shows the difference between treatments for a given comparison between time points. The “median RAD difference” is the estimated value of the median difference between the RAD of a treatment subject and the RAD of a placebo subject. A positive median RAD indicates a greater increase in the relative abundance of *Lactobacillus* spp. from time point 1 to time point 2 in the placebo subjects than in the treatment subjects (or a smaller decrease). An effect of the treatment could not be observed for any of the time point comparisons. Differences in RAD values between the treatment and placebo groups were small, and none were statistically significant.

After having documented the temporal dynamics throughout the study and menstrual cycle, we then assessed the differences between the oral yeast probiotic and placebo groups. Over the 4-week supplementation phase, S. cerevisiae CNCM I-3856 was detected by culture in vaginal samples from 6 of the 34 women (18%) (versus 1/18 women in the placebo group) and in 28 out of 34 (82%) (versus 0/18 in the placebo group) of the investigated fecal samples from the probiotic group, as reported in more detail previously (36). In this study, we evaluated the impact of the oral yeast probiotic on the whole vaginal community using vaginal swabs. Regarding the effects on the entire community composition, no significant differences were found between the probiotic and placebo groups for particular visits based on Bray-Curtis distances (Fig. S3). Overall, when comparing the samples from the placebo and probiotic groups, we found that, on average, the samples from the probiotic group showed higher relative abundances of lactobacilli, with a statistically significant difference at D21 (*P* = 0.04) (Fig. 3b), and higher estimated absolute abundances, which were statistically significant at D7 (*P* = 0.01) (Fig. 3d). The difference in the change in the relative abundance from one time point to another between the probiotic and placebo groups was overall quite limited and never statistically significant (−3.12% to +3.26%) (Fig. 4). Regarding community composition, the L. crispatus*-*dominated samples were highly prevalent in the placebo group, in 50 of 101 samples (50%) (combined samples over all visits), and less so in the probiotic group (65/200 samples [33%]) (Fig. 2b). In comparison, 86 of 200 samples (43%) from the probiotic group were found to be *L. iners* dominated. This dominance was found much less frequently in the placebo group (14 of 101 samples [14%]) (Fig. 2b). Seventeen of 101 placebo samples (17%) and 24 of 210 probiotic treatment samples (12%) showed a combined profile with both *L. iners* and L. crispatus. L. gasseri, L. jensenii, or *L. pasteurii* dominated 3 of the placebo samples (3/101 [3%]) and 7 of the treatment samples (7/200 [4%]). Seventeen of the placebo samples (17%) and 18 of the probiotic treatment samples (9%) were allocated to the non-*Lactobacillus*-dominated group. Additionally, the alpha diversity, as indicated by the inverse Simpson index, was slightly lower in the probiotic treatment group (medians of 1.23 for the treatment group and 1.61 for the placebo group), although both groups showed similar bacterial richness (Fig. 2c; Fig. S4). Most importantly, the differences described here were generally already present in the baseline samples and thus do not necessarily reflect a potential effect of the probiotic intervention.

10.1128/msphere.00239-22.2 FIG S2 Grouping of all of the samples by dominant community members as shown in Fig. 2. Groups 5a, 5b, 5c, and 5d were combined as the “non-*Lactobacillus*-dominated” group. Download FIG S2, TIF file, 0.3 MB. Copyright © 2022 Oerlemans et al. 2022 Oerlemans et al. https://creativecommons.org/licenses/by/4.0/ This content is distributed under the terms of the Creative Commons Attribution 4.0 International license.

10.1128/msphere.00239-22.3 FIG S3 Differences in communities per visit, with each sample compared to the visit 1 sample from the same participant. (a) Beta diversity, as estimated by the Bray-Curtis distance, based on the entire community composition. (b) Change in bacterial richness. (c) Beta diversity, as estimated by the Bray-Curtis distance, based on the *Lactobacillus* community. (d) Change in bacterial richness specific for *Lactobacillus* taxa. (e) Change in alpha diversity based on the inverse Simpson index. Download FIG S3, TIF file, 0.3 MB. Copyright © 2022 Oerlemans et al. 2022 Oerlemans et al. https://creativecommons.org/licenses/by/4.0/ This content is distributed under the terms of the Creative Commons Attribution 4.0 International license.

10.1128/msphere.00239-22.4 FIG S4 Alpha diversity for each sample in terms of the inverse Simpson index (left) and bacterial richness (right). Download FIG S4, TIF file, 0.2 MB. Copyright © 2022 Oerlemans et al. 2022 Oerlemans et al. https://creativecommons.org/licenses/by/4.0/ This content is distributed under the terms of the Creative Commons Attribution 4.0 International license.

### Impact of menstruation and probiotic treatment on potential pathogens.

**FIG 5:**
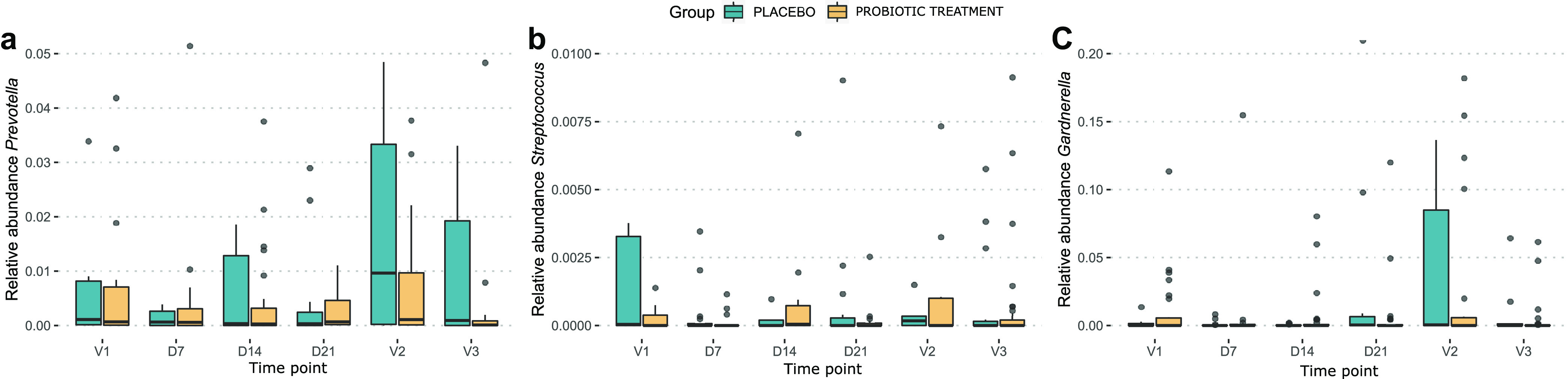
Box plots showing the relative abundances of *Prevotella* (a), *Gardnerella* (b), and Streptococcus (c) throughout the study, separated for the probiotic (blue) and placebo (yellow) groups.

In addition to the community- and dominant-taxon-level analyses of the probiotic treatment, we also analyzed the impacts of the oral yeast probiotic and menstruation on specific taxa of potential pathogens or pathobionts, such as members of the genera *Gardnerella*, *Prevotella*, and Streptococcus (Fig. 5). In the placebo group, the relative abundance of *Prevotella* showed a remarkable increase at V2 (time point shortly after menstruation and at cessation of the study medication) (Fig. 5a). In contrast, in the probiotic group, the abundances of *Prevotella* spp. remained more stable, although this did not result in a significant difference between the probiotic and placebo groups. For the genera *Gardnerella* and Streptococcus, no significant differences between the probiotic and placebo groups were observed, but both showed a specific temporal trend where their relative abundances were highest at V1 and V2, time points that followed menstruation (Fig. 5b and c). However, it is important to mention that these relative abundances were generally quite low.

## DISCUSSION

Although crucial for the health of women and their children, we have yet to determine how to specifically modulate the vaginal microbial community. Here, we investigated the impacts of the menstrual cycle and an oral yeast probiotic treatment on the vaginal microbiome. Overall, the profiles of the samples in this study of 52 healthy women resembled vaginal microbiome profiles described previously, more precisely, with high abundances of *Lactobacillus* species in most vaginal samples (2) and few yeasts (26). Even during oral supplementation with a probiotic yeast, the concentration of yeasts in our samples remained low. This suggests that even though culture indicated some transmission to the vagina of this orally supplemented yeast in some women, the concentrations are low. Therefore, if the desired beneficial activities are dependent on a high concentration of yeast and/or a direct interaction between the yeast and the vaginal mucosa or microbiota, topical application should be considered. However, an indirect effect of the orally supplemented yeast on the vaginal microbiome, e.g., through immunomodulation (34), should not be dismissed, along with the possibility that even at low concentrations, the supplemented yeast might have a relevant beneficial effect, in accordance with the concept of keystone microbes (39) and as our group has observed in other niches (40, 41).

Regarding the vaginal bacterial community, we found that *L. iners* and L. crispatus were very well represented in our cohort. In agreement, previous studies reported vaginal microbial communities dominated by one of these two taxa to be the most prevalent (5–7). However, we also found that these taxa occurred together in various samples, deviating from the traditional view of a single dominant taxon in *Lactobacillus-*dominated vaginal microbiomes, as also recently described (6). Besides the cooccurrence of L. crispatus and *L. iners*, we also encountered samples where L. gasseri and L. crispatus occurred together.

The studied women often showed a certain variability in the vaginal microbiome, as shifts in community profiles were common, with 69% of participants showing at least one shift in their vaginal microbiome. These shifts occurred especially when two *Lactobacillus* species were codominant. At two time points, both shortly after menstruation, the relative abundances of lactobacilli in general, *Gardnerella*, and Streptococcus were altered. Menstruation especially impacted L. crispatus, resulting in lower abundances of this taxon. Although other studies found increases in *L. iners* abundances during menstruation (42, 43), in our study, these abundances remained relatively stable. As L. crispatus is most strongly associated with vaginal health and, on the other hand, the genus *Gardnerella* is known mostly for its association with bacterial vaginosis, it seems that menstruation can potentially make the vaginal community more vulnerable, in agreement with a review by Greenbaum et al. (38). Taken together, our results corroborate that menstruation is an important destabilizing factor for the vaginal microbiome and a relevant time point for microbiome intervention studies. Such a destabilizing factor could create a window of opportunity, during or shortly after its occurrence, for interventions, such as with probiotics, that require at least temporary colonization, which should be investigated further. However, here, we did not observe a clear effect of the probiotic on the relative abundances of *Gardnerella* spp. and Streptococcus spp., indicating that an approach different from the one described here would be advised for interventions aimed at these taxa. Additionally, as menstruation impacts L. crispatus and *L. iners* abundances differently, it is plausible that (probiotic) intervention would also influence communities dominated by these taxa differently. In our study, we have found higher relative abundances of lactobacilli in the probiotic group than in the placebo group, which suggests a supportive action of the probiotic treatment. However, we found that L. crispatus- and *L. iners*-dominated profiles were asymmetrically distributed across the probiotic and placebo groups at the onset of the study, and the relative abundances of lactobacilli were higher for the probiotic group at the time point before intervention (but not statistically significant). This made it difficult to distinguish whether there was an effect of the probiotic treatment or whether this should be attributed to varying effects of the menstrual cycle. These observations clearly highlight the importance of the early stratification of placebo and treatment groups based on their vaginal microbiome profiles at the start of the study, which, in retrospect, was shown here to be equally as important as (if not more important than) variables such as age and/or method of contraception. This would especially be important for populations where high proportions of non-*Lactobacillus*-dominated profiles are encountered, as in these samples, stronger effects in terms of increases in the abundances of lactobacilli and/or decreases in the abundances of pathobionts could be observed. By comparing 16S rRNA gene amplicon sequencing profiles and qPCR results, we could also show that species-specific qPCR for the four typical dominant vaginal lactobacilli is a relatively fast, inexpensive, and accurate way to do this (see Fig. S5 in the supplemental material). The estimated absolute abundances of L. crispatus, *L. iners*, L. gasseri, and L. jensenii based on qPCR could predict which *Lactobacillus* taxon would dominate the 16S rRNA gene profile. This could be expanded with the inclusion of some important non-*Lactobacillus* genera such as *Gardnerella*, *Prevotella*, or Streptococcus to identify which samples are not dominated by lactobacilli.

10.1128/msphere.00239-22.5 FIG S5 Comparison of dominant taxa according to 16S rRNA gene sequencing and qPCR. We assigned qPCR samples to the L. crispatus and *L. iners* group if L. crispatus and *L. iners* were the most abundant taxa according to qPCR with similar concentrations for both (<2-fold difference). Download FIG S5, TIF file, 0.1 MB. Copyright © 2022 Oerlemans et al. 2022 Oerlemans et al. https://creativecommons.org/licenses/by/4.0/ This content is distributed under the terms of the Creative Commons Attribution 4.0 International license.

Finally, we also observed a possible effect of the probiotic treatment on the abundances of *Prevotella* spp. The genus *Prevotella* was previously implicated in the vaginal microbiome profile as typical for aerobic vaginitis and bacterial vaginosis, along with a high diversity and a high abundance of *Gardnerella* spp. in the latter (11, 44). In our results, we encountered *Prevotella* spp. often as main community members in our high-diversity samples, which did not have high relative abundances of *Gardnerella* spp. It was recently proposed that *Prevotella* spp. are community members associated with negative health outcomes, more precisely, severe forms of the inflammatory condition aerobic vaginitis and cervical intraepithelial neoplasia (11, 45). Here, we found that although *Prevotella* species abundances increased in the placebo group, they remained more stable in the probiotic treatment groups. This suggests that the probiotic could have a positive effect on the vaginal microbiome by preventing *Prevotella* spp. from becoming more abundant in the community, but such effects would have to be investigated further.

To conclude, as our study was based on healthy participants, the low concentration of yeasts in the healthy vagina did not allow us to evaluate the possible impacts of the yeast probiotic on the vaginal fungal community, while the prevention of such fungal infections is postulated to be a potential benefit. To be able to observe such possible benefits, much larger sample sizes will be needed. Nonetheless, our study highlighted important factors, such as the menstrual cycle, to consider when designing future intervention studies for vaginal microbiome modulation. Although also a shortcoming of our study, our data especially point out that the stratification of women based on their vaginal community type at study onset not only is crucial but also can be relatively easily performed by qPCR with primers specific for known possible dominating taxa. In addition, the concept of cooccurring dominating taxa (at the species and lower taxonomic levels) should be further explored as this can shine more light on the transition from one community type to another. Currently, there is contradictory evidence regarding the use of probiotics for vaginal health, which might be linked to the interference of the factors that we describe here. Taking factors such as these into account will strengthen studies on microbiome modulation approaches and promote the discovery of treatments for vaginal conditions.

## MATERIALS AND METHODS

### Study design and inclusion of participants.

The analyses described here are part of an exploratory, randomized, double-blind, placebo-controlled, three-parallel-group clinical study, run in a private investigation center (Biofortis Mérieux NutriSciences, Saint-Herblain, France), as described in detail previously by Decherf et al. (36).

After being fully informed of the study risks, benefits, aims, and procedures, 60 women were included and signed an informed-consent form. The study was authorized by the French Health Authority (ANSM), approved by the ethics committee (CPP Nord Ouest III of Caen), and assigned registration number ID-RCB 2017-A02709-44. The study was performed in compliance with the Declaration of Helsinki in accordance with good clinical practice (GCP) standards and current French regulations. The study was registered at ClinicalTrails.gov (identifier NCT03574844).

Three groups were included in the study, a placebo group and two probiotic groups, to which 20 participants each were allocated. Three participants were excluded from the clinical study (36). Five other participants did not agree to the secondary use of their samples in this study and were not included here. The oral probiotic supplement evaluated in the study was provided by Gnosis by Lesaffre (business unit of Lesaffre Group, France), which is composed of 100% Saccharomyces cerevisiae CNCM I-3856, a proprietary and patented strain of Lesaffre registered in the French National Collection of Microorganisms (CNCM). Participants in the two probiotic groups were supplied with two different doses of the probiotic supplement: 2.5 × 10^9^ CFU daily (500-mg probiotic group) and 5 × 10^9^CFU daily (1,000-mg probiotic group). The placebo was composed of maize starch and magnesium stearate. All capsules were presented in blisters and manufactured by Pileje Industries, France. Every participant was asked to consume two capsules (two probiotic capsules for the 1,000-mg group, two placebo capsules for the placebo group, and one capsule of each for the 500-mg group) each day, just before breakfast, with a glass of water. For the analyses presented here, the two probiotic treatment groups were combined.

### Sample collection and DNA extraction.

Vaginal swabs were collected at regular time points: just after menstruation and at the initiation of supplementation (V1), at days 7, 14, 21, 28, and 42 (±2 days). A dry swab (FLOQSwabs; Copan) was used for sample collection and stored at −80°C. Total DNA was extracted from the swab using the phenol-chloroform method as previously described (46), with minor modifications. More precisely, the protocol was adapted for the use of vaginal swabs, which were placed directly into lysis buffer, with minor modifications of the volumes used. The final DNA sample was divided into two aliquots. DNA was quantified using a Qubit fluorometer (Thermo Fisher). One aliquot was used for targeted quantitative real-time PCR (qPCR) to assess changes in the populations of L. crispatus, *L. iners*, L. gasseri, and L. jensenii using the ABI 7500 real-time PCR system, as described previously (36, 47). The other aliquot was transported on dry ice from the clinical research center to the laboratory of Sarah Lebeer (UAntwerp, Antwerp, Belgium) for amplicon sequencing analysis.

### Quantitative real-time PCR.

qPCR analysis to estimate the absolute abundances of members of the microbiota was performed at the clinical research center as described previously (36, 47). Briefly, PCRs were performed in a total volume of 20 μL containing 5 μL DNA, primers (0.5 μM), and probes (0.25 μM). Primer and probe sequences can be found in Table 1. qPCR was performed with the ABI 7500 real-time PCR system. Cycling conditions (40 cycles) were as follows: a denaturation step at 95°C for 15 s and annealing/amplification at 55°C for 1 min. Threshold cycle (*C_T_*) values of samples were compared against a standard curve of DNA of the target taxon.

**TABLE 1:** Primer sequences for qPCR analysis and amplicon sequencing^*a*^

PCR target	Primer direction or probe	Sequence
qPCR		
*L. iners*	Forward	AGTCTGCCTTGAAGATCGG
	Probe	FAM-CCAAGAGATCGGGATAACACCT
	Reverse	CTTTTAAACAGTTGATAGGCATCATC

L. crispatus	Forward	AACTAACAGATTTACTTCGGTAATGA
	Probe	ROX-CCCATAGTCTGGGATACCACTT
	Reverse	AGCTGATCATGCGATCTGC

*Lactobacillaceae*	Forward	ATGGAAGAACACCAGTGGCG
	Reverse	CAGCACTGAGAGGCGGAAAC


Amplicon sequencing (1 example; altered for other barcodes)		
16S rRNA gene V4 region	Forward	CAAGCAGAAGACGGCATACGAGATAACTCTCGAGTCAGTCAGCCGGACTACHVGGGTWTCTAAT
	Reverse	AATGATACGGCGACCACCGAGATCTACACATCGTACGTATGGTAATTGTGTGCCAGCMGCCGCGGTAA

ITS2	Forward	AATGATACGGCGACCACCGAGATCTACACATCGTACGGCCGGTCGAGTAGTGAATCATCGAATCTTTGAA
	Reverse	CAAGCAGAAGACGGCATACGAGATAACTCTCGAGTCAGTCAGGGTCCTCCGCTTATTGATATGC

a Primer sequences for qPCR analysis were obtained from previous studies by Kusters et al. (47) and Zariffard et al. (51), and amplicon sequencing was performed as described by Oerlemans et al. (24), adapted from methods described previously by Kozich et al. (52). FAM, 6-carboxyfluorescein; ROX, carboxyrhodamine.

### Amplicon sequencing analysis and bioinformatics.

Amplicon sequencing targeting the 16S rRNA gene and the ITS2 region to characterize the bacterial and fungal communities in our samples, respectively, was performed as described previously (24). Totals of 2 μL and 5 μL of the received DNA were used for 25 and 30 cycles of PCR amplification (95°C for 20 s, 55°C for 15 s, and 72°C for 1 min for each cycle) with barcoded primers specific for the V4 region of the 16S rRNA gene and the ITS2 region, respectively (Table 1). The result of the PCR was evaluated by loading the product onto a 1% agarose gel. Only a few reactions with the ITS2 primers yielded PCR products with high DNA concentrations (>10 ng/μL), even after multiple PCR attempts. All PCR products of the 16S rRNA PCRs (2 per sample) and at least one PCR product per sample for ITS2 (therefore including ones that did not show a visible product on the gel) were purified (AMPure XP PCR purification; Beckman Coulter), after which their DNA concentrations were measured. The purified PCR products were then pooled equimolarly per target gene, with a maximum of 384 samples for each library, resulting in two 16S rRNA libraries and one ITS2 library. The libraries were sequenced on separate Illumina MiSeq runs (v2 chemistry 2× 250-bp kit; Illumina). Due to the low DNA concentrations in the ITS2 library, the run was not successful, and data were unavailable. Raw data from the 16S rRNA gene runs were subjected to quality control and read processing, performed with the R package DADA2, version 1.6.040 (48). This included forward-reverse-read merging and the removal of reads with conflicting bases and chimeric sequences. Classification of ASVs was performed from the kingdom to the subgenus levels based on EzBioCloud (49). Further processing of reads, including ASV table processing and metadata annotation, was based on the in-house-developed R package tidyamplicons, publicly available at https://github.com/SWittouck/tidyamplicons. Sequencing data are available at the European Nucleotide Archive under accession number PRJEB33108.

### Biostatistical analysis.

The relative abundances of lactobacilli were compared between time points as follows. For a given comparison of two time points, the relative abundance difference (RAD) of the genus *Lactobacillus* between the time points was determined for each participant. The median of these RAD values across participants was then calculated. Statistical significance for each time point comparison was determined with a Wilcoxon signed-rank test on the relative abundance values; the resulting matrix of *P* values was corrected for multiple testing using the method of Benjamini and Yekutieli (50). The effect of treatment on the relative abundance of lactobacilli was assessed similarly. For each time point comparison, the *Lactobacillus* RAD values were compared between the treatment and placebo groups. The effect size was measured by the median of all pairwise differences of RAD values between treatment groups (Hodges-Lehmann estimator), while statistical significance was assessed using a Wilcoxon rank sum test on the RAD values. The *P* value matrix was corrected for multiple testing using the method of Benjamini and Yekutieli (50). For comparisons of relative and estimated absolute abundances between the placebo and treatment groups, Kruskal-Wallis tests were used. For the evaluation of the temporal dynamics in our study, samples were divided into groups according to their dominant taxa to reduce the complexity of the data (Figure S2, Figure S6). A sample was classified as dominated by a taxon if its relative abundance exceeded 40% and it was the most abundant taxon in the sample. As many samples showed high relative abundances of both L. crispatus group ASVs and *L. iners* group ASVs, these were allocated to a separate group if the relative abundances of both taxa exceeded 10% and their combined relative abundance exceeded 50%. Samples not dominated by *Lactobacillus* were combined in a non-*Lactobacillus*-dominated group.

### Data availability.

Sequencing data are available at the European Nucleotide Archive under accession number PRJEB33108.

10.1128/msphere.00239-22.6 FIG S6 Community compositions of all samples from the placebo (a) and probiotic treatment (b) groups, ordered by participant (headers above the graphs) and visit (*x* axis). The *y* axis indicates the relative abundance of each taxon. Download FIG S6, TIF file, 1.8 MB. Copyright © 2022 Oerlemans et al. 2022 Oerlemans et al. https://creativecommons.org/licenses/by/4.0/ This content is distributed under the terms of the Creative Commons Attribution 4.0 International license.
