# Supplementary figures and images for: Impacts of Menstruation, Community Type, and an Oral Yeast Probiotic on the Vaginal Microbiome
Source: mSphere. 2022 Sep 14;7(5):e00239-22. doi: 10.1128/msphere.00239-22 (PMC9599324; doi:10.1128/msphere.00239-22)

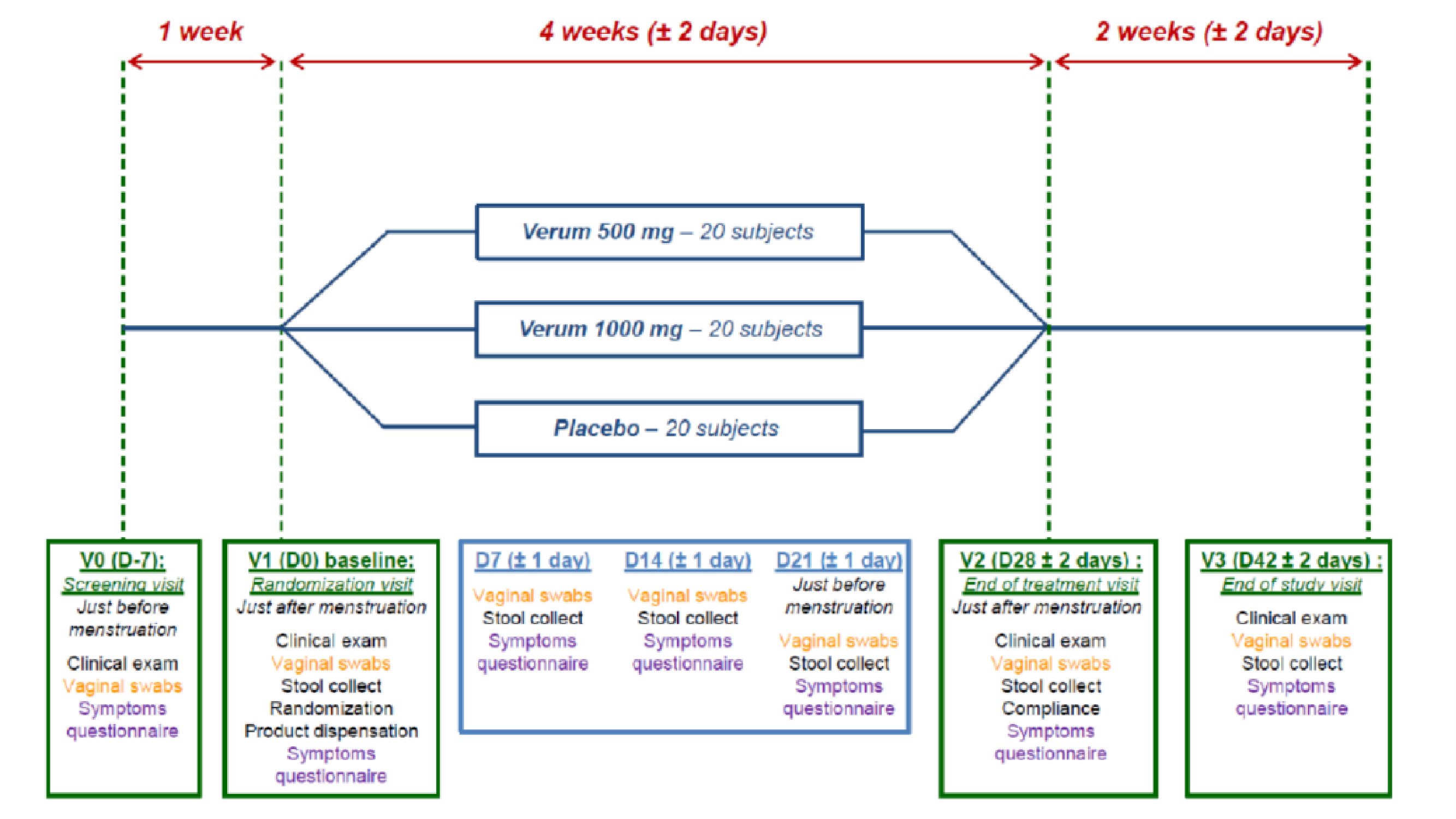

Supplement: FIG S1 [file msphere.00239-22-s0006.tif]

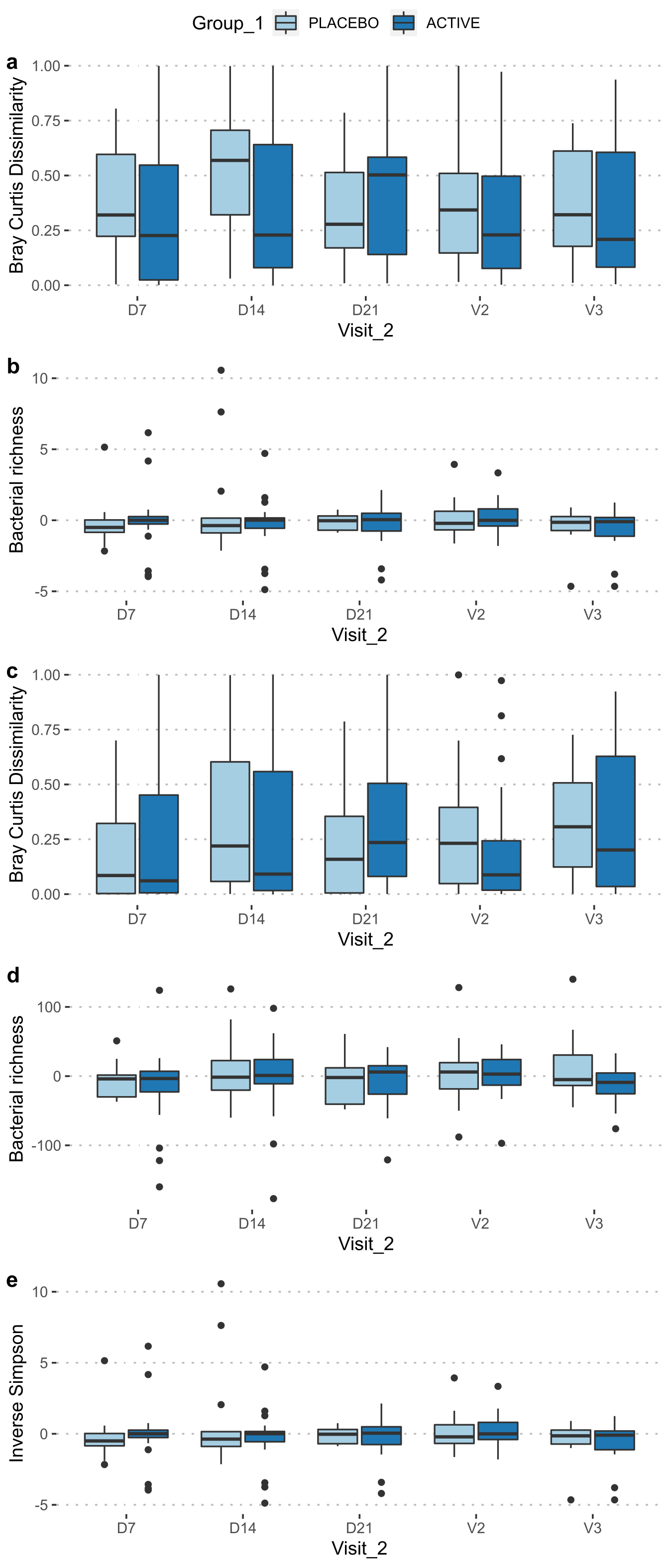

Supplement: FIG S3 [file msphere.00239-22-s0002.tif]

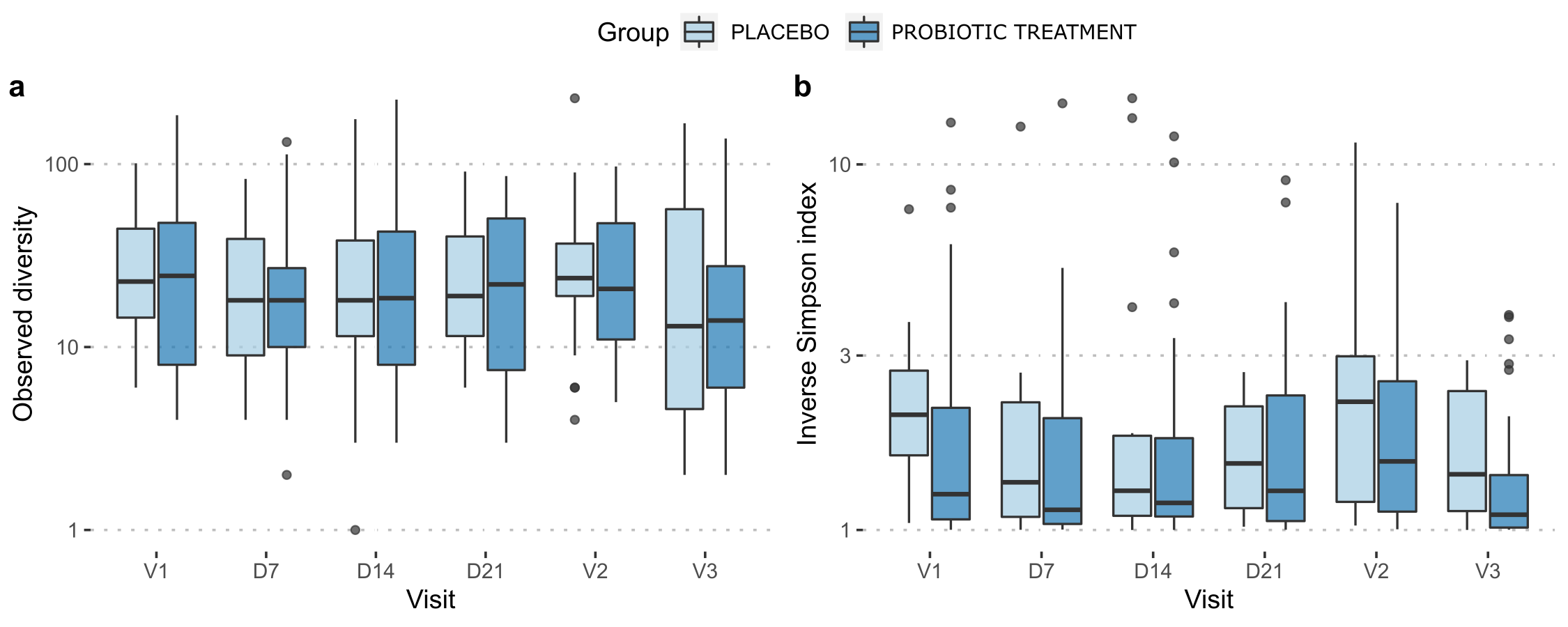

Supplement: FIG S4 [file msphere.00239-22-s0003.tif]

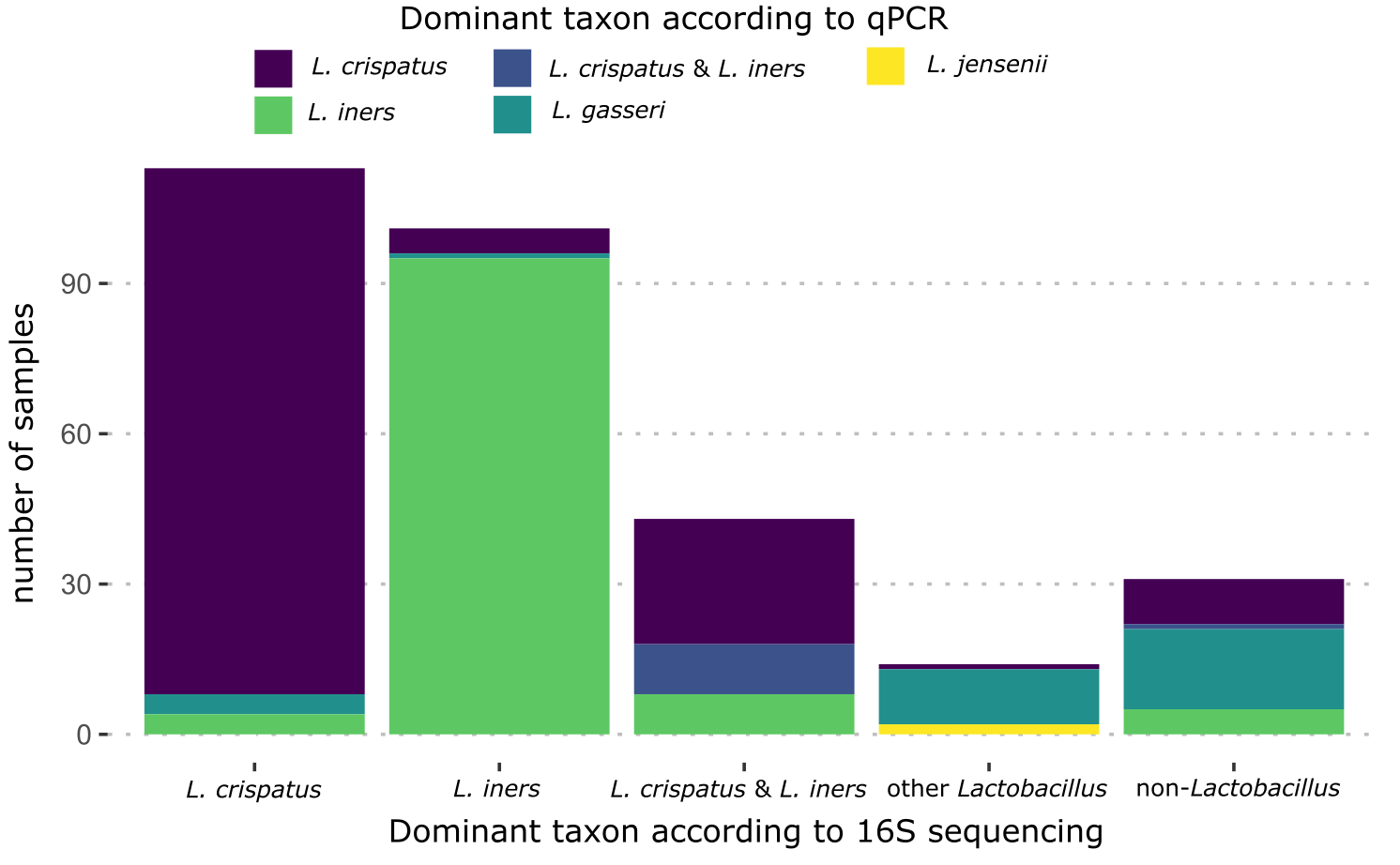

Supplement: FIG S5 [file msphere.00239-22-s0004.tif]

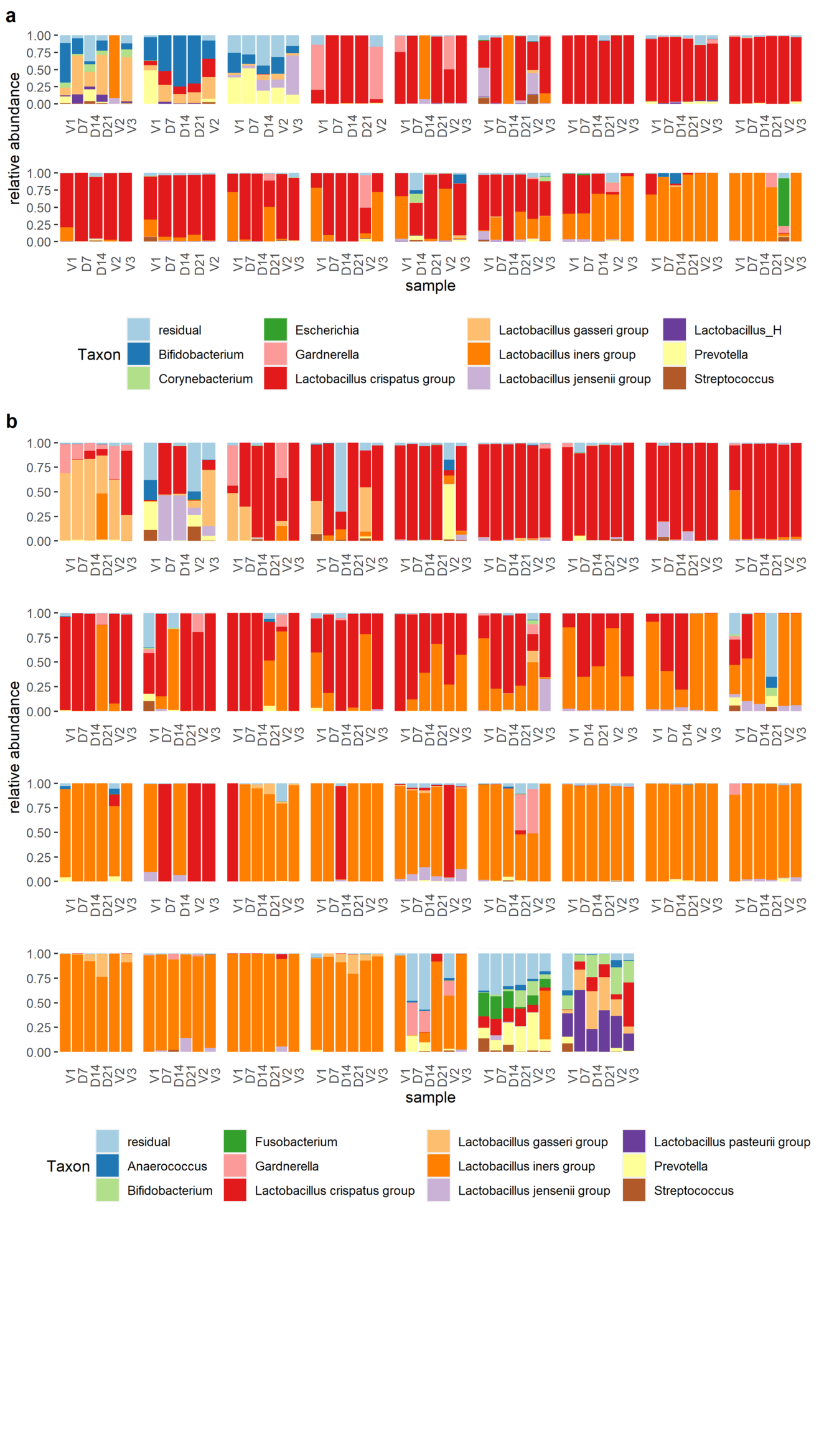

Supplement: FIG S6 [file msphere.00239-22-s0005.tif]
